# Death’s Doings

**Published:** 1865

**Authors:** 


### Editorial.

### DEATH'S DOINGS.

Within the last few days the people of this nation have passed through such a scene and trial as has never before been the lot of this, or perhaps any other nation. The death of our much esteemed and greatly beloved President cast a pall of gloom and sorrow over the people, darker and thicker by far than any or all untoward circumstances that have occurred. It was a stroke hard to bear—a cup whose contents were bitter indeed. Scarcely ever was a ruler so much beloved by his people; and so universal was this that even his enemies could not bring a railing accusation against him: they even wept bitter tears when he fell. He had love for all; mercy was his crowning characteristic. He was the inflexible opponent of that which is wrong, and the lover of that which is good. In him the needy and down-trodden found a sympathizing and helping friend. This Providence to us, thus far, is inscrutable, and, with another, we may say, "He made darkness his secret place; his pavillion round about him were dark waters and thick clouds of the skies." But God will teach us, if we seek to know, It is far better to recognize and acknowledge His operations with a cheerful faith and reliance than to sit down enveloped in a sullen gloom and sadness. ABRAHAM LINCOLN *is dead*; a nation weeps; the poor of the *world* mourn.

